# Expression Analysis of Canine CMTM6 and CMTM4 as Potential Regulators of the PD-L1 Protein in Canine Cancers

**DOI:** 10.3389/fvets.2020.00330

**Published:** 2020-06-11

**Authors:** Hiroto Takeuchi, Satoru Konnai, Naoya Maekawa, Erina Minato, Yoshiki Ichikawa, Atsushi Kobayashi, Tomohiro Okagawa, Shiro Murata, Kazuhiko Ohashi

**Affiliations:** ^1^Department of Disease Control, Faculty of Veterinary Medicine, Hokkaido University, Sapporo, Japan; ^2^Department of Advanced Pharmaceutics, Faculty of Veterinary Medicine, Hokkaido University, Sapporo, Japan; ^3^Department of Clinical Sciences, Faculty of Veterinary Medicine, Hokkaido University, Sapporo, Japan

**Keywords:** canine, CMTM6, CMTM4, PD-L1, molecular identification, cancer immunotherapy

## Abstract

Cancer is one of the most significant causes of death in dogs. Antibody drugs targeting the PD-1/PD-L1 axis represent a promising immunotherapy for both human and canine cancers. However, the regulation mechanisms of PD-L1 expression in canine cancers require further investigation to better understand the resistance mechanisms to anti-PD-L1 therapy. Recent reports have shown that CMTM6 and CMTM4 are critical regulators of PD-L1 protein expression in human cancer cells. By preventing PD-L1 from lysosome-mediated degradation, CMTM6 maintains PD-L1 expression on the cell surface. However, the literature has not reported on CMTM6 and CMTM4 in dogs, and their functions are completely unknown. To reveal a regulation mechanism of PD-L1 in canine cancers, this study firstly identified the gene sequences of *CMTM6* and *CMTM4*. Then, the expression analysis of these proteins was performed by immunohistochemistry. Furthermore, the functions of CMTM6 and CMTM4 in regulating PD-L1 expression were examined by gene knockdown of *CMTM6* and *CMTM4*. Canine CMTM6 and CMTM4 displayed high amino acid sequence identities compared with those of humans and mice. An immunohistochemical analysis using cross-reactive antibodies revealed that canine malignant melanoma and osteosarcoma express CMTM6, CMTM4, and PD-L1 simultaneously. Gene knockdown of *CMTM6* and *CMTM4* with RNA interference significantly reduced the cell surface expression of PD-L1 in a canine cell line. These results suggest that CMTM6 and CMTM4 are regulators of PD-L1 expression in canine cancers and could serve as potential therapeutic targets to enhance antitumor immunity.

## Introduction

The importance of cancer treatment has recently been highlighted in dogs because tumor-associated deaths comprise approximately 40% of canine mortality (1). So far, surgery, chemotherapy, and radiation have been the main treatment modalities for canine cancers. But, in some cases, treatment options can be limited because of the anatomical sites, sensitivity to chemotherapy or radiotherapy, and/or adverse effects of the treatment. Therefore, the development of novel treatment modalities is strongly desired in order to provide better veterinary care for canine patients.

Immunotherapy has become the fourth pillar of cancer treatment in human medicine largely due to the success of immune checkpoint inhibitors (ICIs). Programmed death 1 (PD-1), an immune checkpoint molecule, is an inhibitory receptor that belongs to the CD28 family. PD-1 is mainly expressed on activated T cells and transmits suppressive signals into the lymphocytes, resulting in the attenuation of antigen-specific immune responses (2–5). A ligand for PD-1, programmed death ligand 1 (PD-L1), can be expressed on tumor cells; thus, the PD-1/PD-L1 axis is involved in the immune evasion of tumor cells (6, 7). Importantly, this suppression is reversible, and the blockade of the PD-1/PD-L1 pathway using anti-PD-1 or PD-L1 antibodies can restore the effector functions of T cells including the capacities for cell proliferation and cytokine secretion (8, 9). In human clinical trials, antibody therapies targeting the PD-1/PD-L1 pathway showed tolerable safety profiles and promising antitumor activities (10, 11), and now these antibody drugs are widely used to treat various cancers in clinics.

In our previous study, we found that PD-1 was highly expressed on tumor-infiltrating lymphocytes obtained from canine oral melanoma. Moreover, PD-L1 was expressed in many cancer types including canine oral melanoma, osteosarcoma, and mast cell tumors (12, 13). These results suggest that the PD-1/PD-L1 axis is involved in the immune evasion of canine cancers. Recently, we established a canine chimeric anti-PD-L1 monoclonal antibody and conducted a pilot clinical study in dogs with oral malignant melanoma and undifferentiated sarcoma. The anti-PD-L1 therapy induced substantial tumor regression in a subset of patients with these cancer types. However, most patients did not have an objective response despite identifying PD-L1 expression in the tumor cells by immunohistochemistry (14). Therefore, the identification of biomarkers that can predict patient subpopulations will benefit anti-PD-L1 antibody therapy. The understanding of the molecular mechanisms of treatment resistance is urgently required.

The elucidation of detailed mechanisms that regulate PD-L1 expression is of particular importance to obtain insights into the resistance mechanisms to anti-PD-L1 antibody therapy. Recent studies on human cancer cell lines have demonstrated that CKLF-like MARVEL transmembrane domain-containing protein 6 (CMTM6) is a critical regulator of the PD-L1 protein (15, 16). CMTM6 is a ubiquitously expressed, type 3 transmembrane protein that co-localizes with PD-L1 in the plasma membrane and recycling endosomes. CMTM6 maintains PD-L1 cell surface expression by preventing PD-L1 from being degraded in lysosomes (15). CMTM4, another CMTM family member that is closely related to CMTM6, is also identified as a positive regulator of PD-L1 in the absence of CMTM6 (16). Because the depletion of CMTM6 enhanced tumor-specific T cell responses by reducing PD-L1 expression (15), CMTM6 has gained attention not only as a key molecule involved in the PD-1/PD-L1 axis but also as a potential therapeutic target against cancers.

However, no reports are available on CMTM6 or CMTM4 in dogs, and their association with PD-L1 requires further examination. To reveal the regulation mechanism of PD-L1 in canine cancers, this study firstly identified *CMTM6* and *CMTM4* mRNA open reading frame (ORF) sequences. Then, CMTM6, CMTM4, and PD-L1 protein expressions were evaluated in canine cancers by immunohistochemistry. Finally, the role of CMTM6 and CMTM4 in PD-L1 regulation was examined in a canine cell line by inducing CMTM6 and CMTM4 knockdown.

## Materials and Methods

### Canine Samples

The use of animal samples throughout this study was approved by the Institutional Animal Care and Use Committee (#15-0149), Faculty of Veterinary Medicine, Hokkaido University, which has been fully accredited by the Association for Assessment and Accreditation of Laboratory Animal Care International. Peripheral blood samples were obtained from healthy beagles kept at the Experimental Animal Facility, Faculty of Veterinary Medicine, Hokkaido University. Tumor samples were surgically excised from the dogs at the Veterinary Teaching Hospital, Faculty of Veterinary Medicine, Hokkaido University.

### Cell Culture

Canine melanoma cell lines LMeC (17), CMeC (18), CMM-1 (19), and CMM-2 (19) and canine osteosarcoma cell lines POS (17) and HMPOS (20) were cultured in RPMI 1640 medium (Sigma-Aldrich, St. Louis, MO, USA) supplemented with 10% inactivated fetal bovine serum (FBS) (Thermo Fisher Scientific, Waltham, MA, USA), 2 mM L-glutamine (Thermo Fisher Scientific), 200 μg/mL streptomycin (Thermo Fisher Scientific), and 200 U/mL penicillin (Thermo Fisher Scientific) at 37°C and 5% CO_2_. A canine macrophage-monocyte cell line DH82 (ATCC CRL-10389) (21) was cultured in EMEM medium (ATCC, Manassas, VA, USA) supplemented with 15% inactivated FBS at 37°C and 5% CO_2_. Canine peripheral blood mononuclear cells (PBMCs) were isolated from peripheral blood samples by density gradient centrifugation on Percoll (GE Healthcare, Little Chalfont, UK) and were cultured in RPMI 1640 medium supplemented with 10% inactivated FBS, 2 mM L-glutamine, 200 μg/mL streptomycin, and 200 U/mL penicillin at 37°C and 5% CO_2_. To stimulate the PBMCs, 20 ng/mL PMA (Sigma-Aldrich) and 1 μg/mL ionomycin (Sigma-Aldrich) were added to the culture medium, and the cells were cultured for 2 h. ExpiCHO-S cells (Thermo Fisher Scientific) were cultured in ExpiCHO Expression Medium (Thermo Fisher Scientific) at 37°C and 8% CO_2_ on an orbital shaker.

### RNA Extraction and cDNA Synthesis

Total RNA was extracted from canine tumor tissues, cancer cell lines, and beagle dog derived PBMCs stimulated with PMA/ionomycin, using TRI reagent (Molecular Research Center, Cincinnati, OH, USA) following the manufacturer's instructions. Residual genomic DNA was digested by DNaseI (Thermo Fisher Scientific) treatment. cDNA was synthesized from 1 μg of the total RNA using PrimeScript Reverse Transcriptase (Takara Bio, Otsu, Japan) and oligo dT primer, following the manufacturer's instructions.

### Identification of Canine *CMTM6* and *CMTM4* Genes

cDNA was synthesized from a beagle dog derived PBMCs stimulated with PMA/ionomycin as described above. Primer pairs for the canine *CMTM6* and *CMTM4* genes were designed based on the predicted dingo *CMTM6* and *CMTM4* mRNA sequences registered in the GenBank database (XM_025461097.1 and XM_025426371.1, respectively). The ORF sequence of canine *CMTM6* and the partial sequence of *CMTM4* were amplified from beagle cDNA by PCR using primer pairs 5′-GAG ACC AGG AAG TGA CGG C-3′ (CMTM6 forward)/5′-TTC CCC TTG CTC TCC AAA AGA A-3′ (CMTM6 reverse) and 5′-TTT GGC CCT GAT TGC GTT CAT C-3′ (CMTM4 inner forward)/5′-GGC CAC TCT GAA AAA CTT TCC C-3′ (CMTM4 reverse), respectively. TaKaRa Ex Taq (Takara Bio) was used as the DNA polymerase. The amplicons were purified using the FastGene gel/PCR extraction kit (Nippon Genetics, Tokyo, Japan) and were cloned into T-Vector pMD20 (Takara Bio). The nucleotide sequences were analyzed using the GenomeLab GeXP Genetic Analysis System (SCIEX, Framingham, MA, USA). 5′-RACE was performed with the 5′-RACE system for rapid amplification of cDNA ends (Invitrogen, Carlsbad, CA, USA) to determine the canine *CMTM4* ORF sequence using a gene-specific primer (5′-AGT TAC TAC AAA TGC ACT AC-3′). Based on the determined sequence, a new forward primer 5′-CGA GGG GAA GCG ATG CGG-3′ (CMTM4 forward) was designed to amplify the full-length ORF sequence. The amplicon was sequenced as described above. The identified sequences were translated and aligned using BioEdit software (22). The percent identities of the deduced amino acid sequences were calculated with the BLAST program (https://blast.ncbi.nlm.nih.gov/Blast.cgi). Conserved domains within canine CMTM6 and CMTM4 were predicted with the CD Search program (https://www.ncbi.nlm.nih.gov/Structure/cdd/wrpsb.cgi). Unrooted neighbor-joining phylogenetic trees were constructed using Mega version 7 software (23, 24).

### Quantification of *CMTM6* and *CMTM4* mRNA by qRT-PCR

qRT-PCR was performed with the QuantStudio 12K Flex Real-Time PCR System (Thermo Fisher Scientific) using TB Green Premix DimerEraser (Takara Bio) according to the manufacturer's instructions. The specific primer pairs for *CMTM6* and *CMTM4* were designed based on the identified sequences (5′-GAG AAT GGA GCG GTG TAC GG-3′ and 5′-GCA GCT GCA GGA CTT TGA AC-3′ for *CMTM6*, 5′-GGT CAA CAC TGG ACT CAG CAC-3′ and 5′-TTC TGC ACT GCC AGG AAT GTG-3′ for *CMTM4*). Relative gene expressions were calculated using the 2^−ΔΔCT^ method. The data were normalized to the expression of an internal control gene, *HPRT1*, which was similarly quantified using primers 5′-TGG CGT CGT GAT TAG TGA TGA-3′ and 5′-CAG AGG GCT ACG ATG TGA TGG-3′.

### Preparation of Canine CMTM6- and CMTM4-Expressing Cells

To construct expression vectors that induce the simultaneous expression of either canine CMTM6 or CMTM4 and *Aequorea coerulescens* green fluorescent protein 1 (AcGFP1), the ORF sequences of canine *CMTM6* and *CMTM4* were amplified by PCR using specific primers containing the *Bgl*II or *EcoR*I restriction sites. The amplicons were digested by the restriction enzymes and were inserted into the multicloning site of the pIRES2-AcGFP1 vector (Takara Bio). These vectors were named pIRES2-AcGFP1-cCMTM6 and pIRES2-AcGFP1-cCMTM4, respectively. For transient expression, ExpiCHO-S cells were transfected with pIRES2-AcGFP1-cCMTM6 or pIRES2-AcGFP1-cCMTM4 using the ExpiFectamine CHO Transfection Kit (Thermo Fisher Scientific) according to the manufacturer's instructions. An empty pIRES2-AcGFP1 vector was used as the mock. The cells were cultured for 48 h after transfection and were subjected to analysis.

### Flow Cytometric Analysis of Canine CMTM6, CMTM4, and PD-L1

To evaluate the cross-reactivities of rabbit polyclonal anti-human CMTM6 and anti-human CMTM4 antibodies, flow cytometry was performed using canine CMTM6- or CMTM4-expressing cells. The cells were fixed and permeabilized using the FOXP3 Fix/Perm Buffer Set (BioLegend, San Diego, CA, USA), and intracellular staining was performed using 10 μg/mL of rabbit polyclonal anti-human CMTM6 (HPA026980; Sigma-Aldrich) or rabbit polyclonal anti-human CMTM4 antibody (LS-C368889; LifeSpan Biosciences, Seattle, WA, USA) at room temperature for 30 min. Rabbit control IgG (SouthernBiotech, Birmingham, AL, USA) was used as the negative control antibody. After incubation with 1 μg/mL of Alexa Fluor 647-conjugated goat F(ab′)_2_ anti-rabbit IgG (H+L) antibody (Thermo Fisher Scientific) at room temperature for 30 min, more than 50,000 cells were analyzed with a FACS Verse flow cytometer (BD Biosciences, San Jose, CA, USA).

Flow cytometry was performed using anti-PD-L1 monoclonal antibody to examine PD-L1 expression on the cell surface (12, 25). The cells were incubated with 10 μg/mL of anti-PD-L1 antibody (6C11-3A11, rat IgG_2a_, Figure S1) at room temperature for 30 min, followed by another incubation with 1 μg/mL of allophycocyanin-conjugated goat anti-rat Ig antibody (SouthernBiotech) at room temperature for 30 min. Rat IgG_2a_ (κ) isotype control (R35-95, BD Biosciences) was used as the negative control antibody. Cell fluorescence in 1,500–20,000 cells of AcGFP1^+^ cells was analyzed as described above.

### Immunohistochemical Analysis of CMTM6, CMTM4, and PD-L1 in Canine Cancers

Formalin-fixed and paraffin-embedded tumor tissues of canine malignant melanoma (*n* = 4) and osteosarcoma (*n* = 4) were cut into 2–4-μm-thick sections, dried on silane-coated slides, and deparaffinized in xylene. For the malignant melanoma samples, the melanin pigments were bleached using 0.25% potassium permanganate and 2% oxalic acid. Antigen retrieval was performed by heating the sections in 0.01 M citrate buffer (pH 6.0). CMTM6 and CMTM4 were stained with 4 μg/mL of anti-human CMTM6 antibody and 10 μg/mL of anti-human CMTM4 antibody, respectively, using the Histofine SAB-PO (MULTI) Kit (Nichirei, Tokyo, Japan). PD-L1 was stained with 5 μg/mL of anti-PD-L1 antibody (6C11-3A11, Figure S1) using the Vectstain Elite ABC Rat IgG kit (Vector Laboratories, Burlingame, CA, USA). Positive staining was visualized with 3,3′-diaminobenzidine tetrahydrochloride. For negative control, the sections were incubated with the isotype control antibodies (rabbit IgG for CMTMs, rat IgG_2a_ for PD-L1). The staining result was evaluated by two experienced pathologists.

### Gene Knockdown of CMTM6 and CMTM4

Small interfering RNAs (siRNAs) for canine *CMTM6* and *CMTM4* were designed by BLOCK-iT RNAi Designer. siCMTM6-1 sense 5′-AGU GUA CUU UGU GUG GAG GAC UUU A-3′, siCMTM6-2 sense 5′-GCA CUC CAG UUU ACA ACC AAG UCG A-3′, siCMTM4-1 sense 5′-AGA UCA ACU GGA ACC UGA CAG AUU U-3′, siCMTM4-2 sense 5′-GCC GUG AUA UUU GGC UUC UUG GCA A-3′ and siCMTM4-3 sense 5′-GGC CCU GAU UGC GUU CAU CAU CUG CAU A−3′ were synthesized (Stealth RNAi siRNA, Thermo Fisher Scientific). A canine macrophage-monocyte cell line DH82 (ATCC CRL-10389) (21) was used for the gene knockdown of CMTM6 and CMTM4. Transfection was performed using 10 pmol/mL (for siCMTM6) or 5 pmol/mL (for siCMTM4) of each siRNA and Lipofectamine 3000 Reagent (Thermo Fisher Scientific). The cells were incubated for 48 h before analysis. Stealth RNAi siRNA Negative Control Med GC Duplex #2 (Thermo Fisher Scientific) was used as the scrambled control. After the incubation, mRNA expression of *CMTM6* and *CMTM4* and protein expression of PD-L1 in the transfected cells was analyzed described above.

### Statistical Analysis

Turkey's multiple comparison test was used, and *p* < 0.05 was considered statistically significant. All analyses were performed using the statistical analysis software JMP 14 (SAS Institute Inc., Cary, NC, USA).

### Nucleotide Sequence Accession Numbers

The nucleotide sequences of the canine *CMTM6* and *CMTM4* genes have been submitted to the DDBJ/EMBL-Bank/GenBank database under accession numbers LC514675 (CMTM6) and LC514676 (CMTM4).

## Results

### *CMTM6* and *CMTM4* Genes Are Conserved Among Mammalian Species Including Dogs

**Figure 1:**
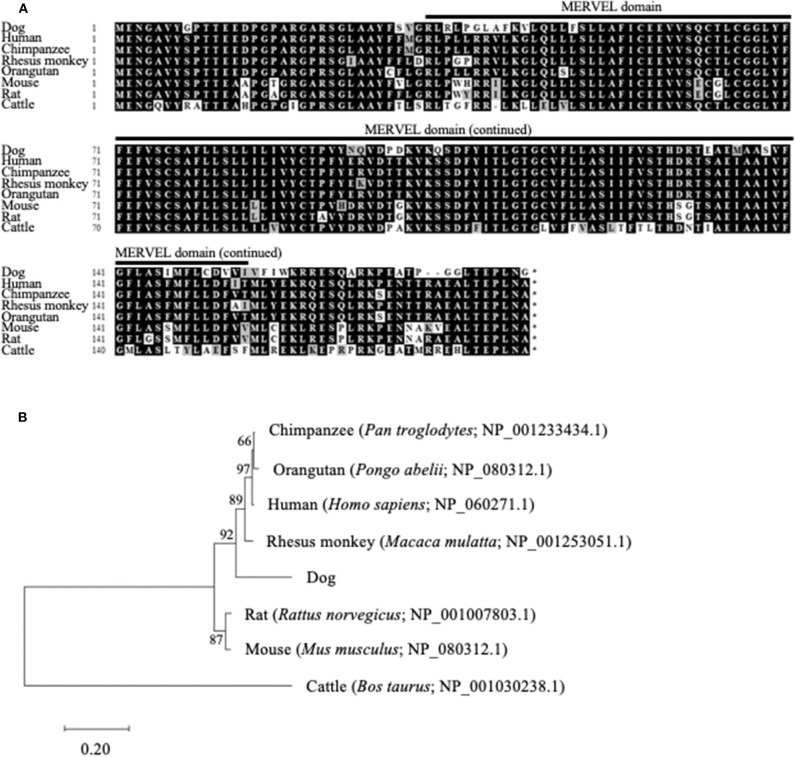
Comparison of the *CMTM6* gene among mammalian species. **(A)** Multiple alignment of the deduced amino acid sequences of CMTM6. The predicted MARVEL domain in canine CMTM6 (33-154) is shown. **(B)** Phylogenetic analysis of canine CMTM6 in relation to those of other mammalian species. The bootstrap consensus tree was inferred from 1,000 replicates. The bootstrap percentages are shown next to the branches. The GenBank accession numbers of each reference sequence are indicated.

**Figure 2:**
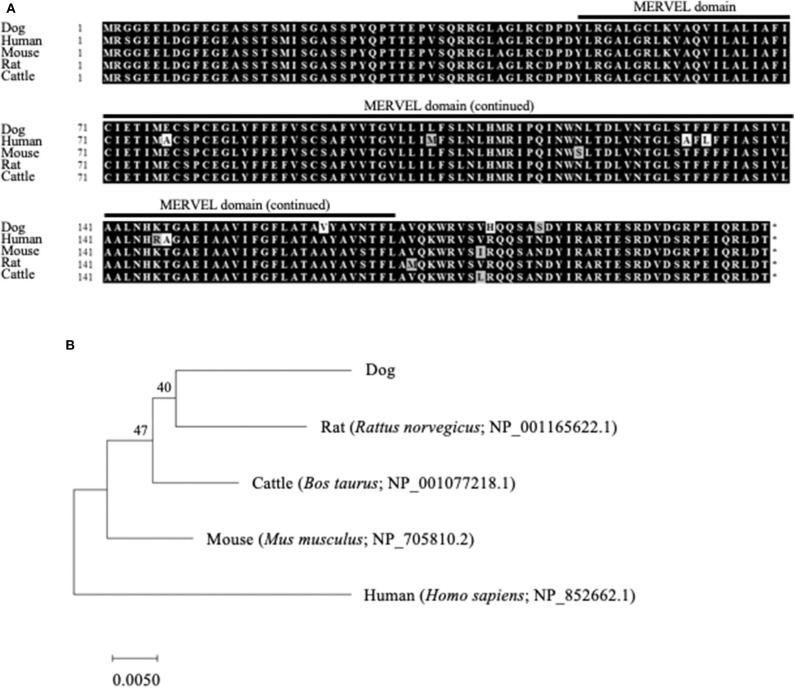
Comparison of the *CMTM4* gene among mammalian species. **(A)** Multiple alignment of the deduced amino acid sequences of CMTM4. The predicted MARVEL domain in canine CMTM4 (49–170) is shown. **(B)** Phylogenetic analysis of canine CMTM4 in relation to those of other mammalian species. The bootstrap consensus tree was inferred from 1,000 replicates. The bootstrap percentages are shown next to the branches. The GenBank accession numbers of each reference sequence are indicated.

The ORF nucleotide sequences of canine *CMTM6* and *CMTM4* were determined using cDNA obtained from beagle PBMCs. The canine *CMTM6* and *CMTM4* ORF sequences were 549 and 627 bp in length and encoded 182 and 208 amino acid polypeptides, respectively. Both canine CMTM6 and CMTM4 contained a predicted functional domain, MAL and related proteins for vesicle trafficking and membrane link (MARVEL) domain; this domain is possibly involved in membrane apposition events in various cellular processes (26) (Figures 1A, 2A). The phylogenetic trees were inferred in relation to the CMTM6 or CMTM4 amino acid sequences of other mammalian species, which were available in the GenBank database (Figures 1B, 2B). These genes appeared to be highly conserved among mammalian species, although the number of species included in the analysis was limited. Canine CMTM6 displayed 78.7 and 73.6% amino acid sequence identities with human and mouse CMTM6, respectively. Canine CMTM4 displayed 93.8% and 96.2% amino acid sequence identities with human and mouse CMTM4, respectively.

### *CMTM6* and *CMTM4* mRNA Is Expressed in Dogs

**Figure 3:**
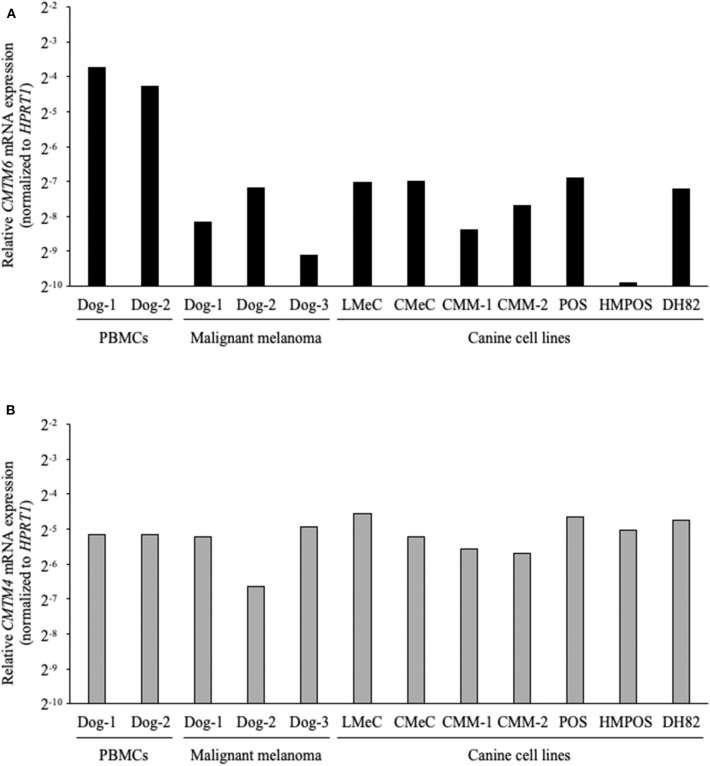
*CMTM6* and *CMTM4* mRNA expression in canine samples. **(A)** *CMTM6* and **(B)** *CMTM4* mRNA expression was quantitated by qRT-PCR in PBMCs from healthy dogs (*n* = 2), malignant melanoma tissues (*n* = 3), melanoma cell lines (LMeC, CMeC, CMM-1, and CMM-2), osteosarcoma cell lines (POS and HMPOS), and a macrophage-monocyte cell line (DH82). The relative gene expressions of *CMTM6* and *CMTM4* were calculated using the 2^−ΔΔCT^ method. *HPRT1* was used as an internal control gene.

Next, the gene expressions of *CMTM6* and *CMTM4* were examined by qRT-PCR in the canine samples. PBMCs from healthy dogs (*n* = 2), malignant melanoma tissues (*n* = 3), melanoma cell lines (LMeC, CMeC, CMM-1, and CMM-2), osteosarcoma cell lines (POS and HMPOS), and a macrophage-monocyte cell line (DH82) were subjected to the analysis. *CMTM6* and *CMTM4* mRNA expression was detectable in all these samples (Figure 3), suggesting that *CMTM6* and *CMTM4* are ubiquitously expressed genes in canine immune cells and cancer cells.

### CMTM6, CMTM4, and PD-L1 Proteins Are Expressed in Canine Cancer Tissues

**Figure 4:**
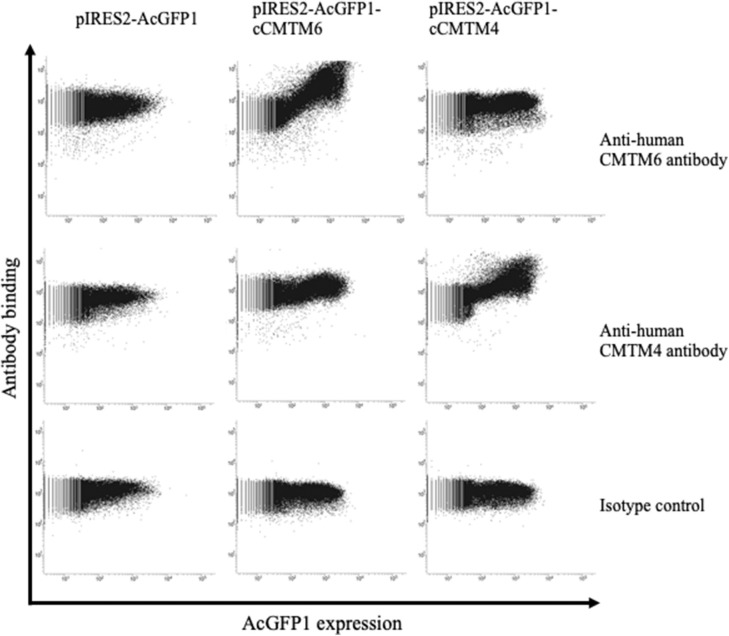
Cross-reactivities of anti-human CMTM6 and CMTM4 antibodies with canine CMTM6 and CMTM4. Binding of anti-human CMTM6 antibody (HPA026980, Sigma-Aldrich) and anti-human CMTM4 antibody (LS-C368889, LifeSpan Biosciences) to canine CMTM6- and CMTM4-expressing cells was examined by flow cytometry. Rabbit IgG was used as isotype-matched control antibody. Using a bicistronic expression vector, AcGFP1 expression was utilized to predict CMTM6 or CMTM4 expression. Antibody binding to the AcGFP1^+^ cell fraction was considered a specific reaction.

To assess the protein expressions of CMTM6 and CMTM4 in canine cancers, we firstly examined the cross-reactivities of commercially available antibodies against human CMTM6 and CMTM4 using canine CMTM6- or CMTM4-expressing cells. To prepare the expression vectors of canine CMTM6 or CMTM4, each identified gene sequence was inserted into a bicistronic expression vector that allows the simultaneous expression of genes of interest and AcGFP1 from the same mRNA transcript. Accordingly, transfectants with AcGFP1 expression are expected to express canine CMTM6 or CMTM4. A flow cytometric analysis of the expressing cells revealed that anti-human CMTM6 and CMTM4 antibodies can detect canine CMTM6 and CMTM4, respectively, because each antibody binding was observed only in the AcGFP1^+^ cells among their corresponding expressing cells (Figure 4).

**Figure 5:**
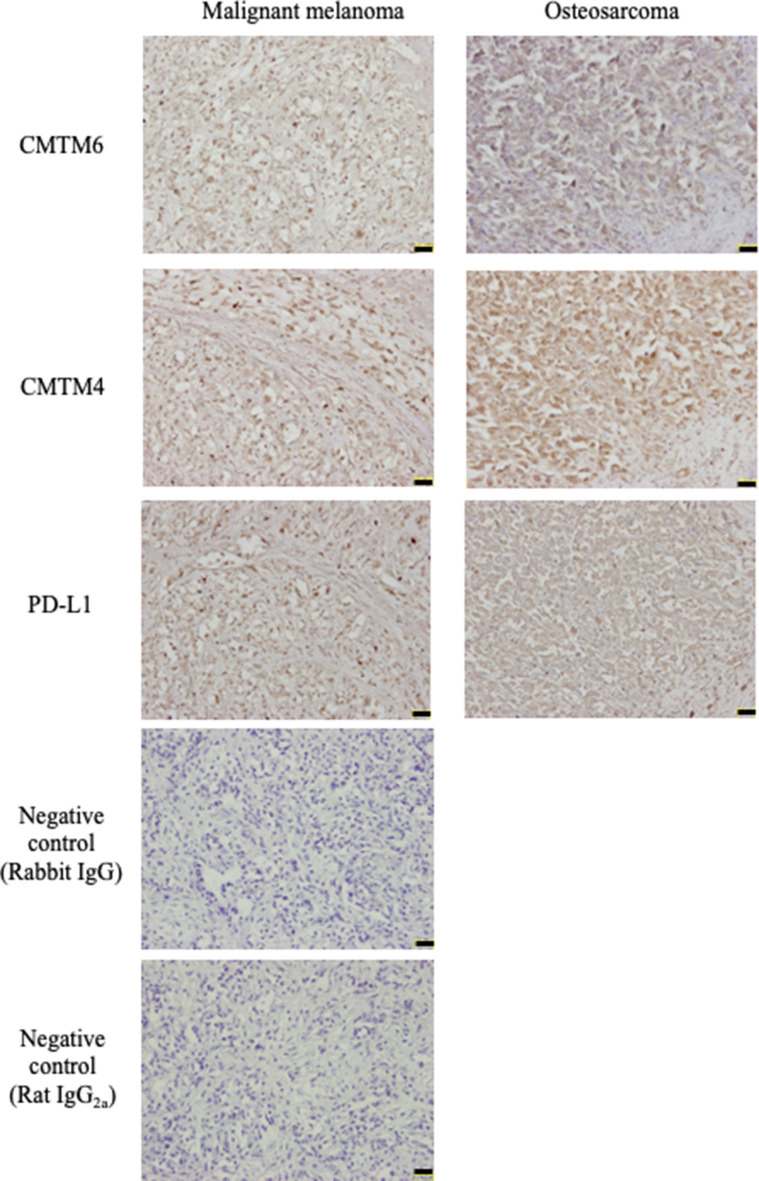
CMTM6, CMTM4, and PD-L1 protein expressions in canine cancers. Canine malignant melanoma (*n* = 4) and osteosarcoma (*n* = 4) samples were analyzed for CMTM6, CMTM4, and PD-L1 expression by immunohistochemistry. For negative control, the sections were incubated with isotype-matched control antibody. Representative results for malignant melanoma (Sample #1) and osteosarcoma (Sample #5) are shown (scale bar: 50 μm).

Subsequently, an immunohistochemical analysis was conducted using these cross-reactive antibodies in canine cancer samples. CMTM6 and CMTM4 expression was found in all malignant melanoma (*n* = 4) and osteosarcoma (*n* = 4) samples. Interestingly, all these cancers also expressed PD-L1. Representative photos in the IHC are shown in Figure 5.

### CMTM6 and CMTM4 Knockdown Downregulates PD-L1 Expression on the Cell Surface in a Canine Cell Line

**Figure 6:**
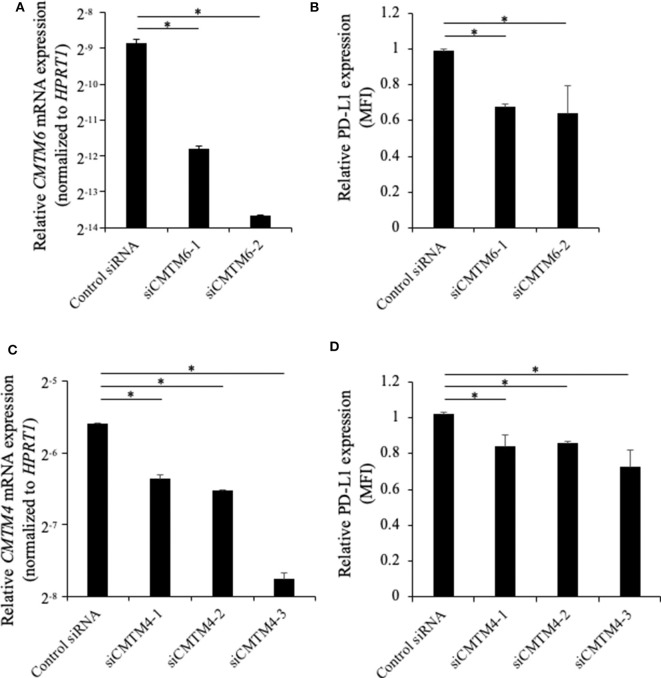
Gene knockdown of CMTM6 or CMTM4 in a PD-L1–expressing canine cell line. DH82 cells were transfected with siRNAs against canine *CMTM6* or *CMTM4* and cultured for 48 h before the analysis. **(A)** The effects of CMTM6 knockdown on *CMTM6* mRNA expression were examined by qRT-PCR. Data are shown as the means ± SD. **(B)** The effects of CMTM6 knockdown on PD-L1 expression was analyzed by flow cytometry. Data are shown as the means of MFI ± SD. **(C)** The effects of CMTM4 knockdown on *CMTM4* mRNA expression were examined by qRT-PCR. Data are shown as the means ± SD. **(D)** The effects of CMTM4 knockdown on PD-L1 expression on the cell surface was analyzed by flow cytometry. Data are shown as the means of MFI ± SD. The statistical analysis was performed using Tukey's test. *p < 0.05.

To investigate the role of canine CMTM6 and CMTM4 in PD-L1 regulation, siRNAs were designed and introduced into a canine cell line, DH82. DH82 expresses PD-L1 constitutively, whereas all other cell lines used in this study do not express PD-L1 without stimulation (12). Both two siRNAs for CMTM6 (siCMTM6-1 and -2) efficiently reduced *CMTM6* mRNA expression (Figure 6A) and significantly decreased the cell surface expression of PD-L1 (Figure 6B). All three siRNAs for CMTM4 (siCMTM4-1, -2, and -3) efficiently reduced CMTM4 mRNA expression (Figure 6C) and significantly decreased the cell surface expression of PD-L1 (Figure 6D). These results indicated that CMTM6 and CMTM4 are possible PD-L1 regulators in canine cells.

## Discussion

ICIs such as anti-PD-1 and anti-PD-L1 antibodies have become a novel treatment option in various cancers affecting humans including malignant melanoma and non-small-cell lung cancer (NSCLC) (27). Anti-PD-L1 antibody has been developed for canine cancer treatment, and its promising efficacy against malignant melanoma and undifferentiated sarcoma was reported in a pilot clinical study (14). However, because only a subset of patients achieves objective responses in both humans and dogs, the identification of predictive biomarkers for treatment outcomes and the investigation of resistance mechanisms to antibody therapy are necessary.

CMTM6 and CMTM4 have recently been identified as critical regulators of PD-L1 protein expression in humans (15, 16). However, their gene sequences have not even been determined experimentally in dogs. To better understand the regulation mechanisms of PD-L1 expression in canine cancers, this study attempted to molecularly characterize canine CMTM6 and CMTM4. The deduced amino acid sequences of canine CMTM6 and CMTM4 were highly similar to their corresponding genes in other mammalian species. Interestingly, the MARVEL domain was also predicted in canine CMTM6 and CMTM4, suggesting that functions of these genes are conserved in dogs. The expression analysis by qRT-PCR revealed that canine *CMTM6* and *CMTM4* can be expressed in canine immune cells and cancer cells, both of which are considered important PD-L1 sources in the suppression of antitumor immunity (28). In the immunohistochemical analysis, PD-L1 expression was found in CMTM6- and CMTM4-expresssing cancers, which supports the hypothesis that canine CMTM6 and CMTM4 are involved in the expression of PD-L1. Finally, CMTM6 and CMTM4 knockdown in a PD-L1–expressing cell line resulted in the downregulation of cell surface PD-L1, demonstrating the potential of CMTM6 and CMTM4 as a key molecule for canine PD-L1 regulation.

PD-L1 expression is known to be regulated at multiple levels by various mechanisms, including genetic alteration, inflammatory signaling, oncogenic signaling, microRNA, and post-translational modification (29). In dogs, IFN-γ stimulation was the only known inducer of PD-L1 protein expression in cancer cells (12) and possibly functioned via the transcriptional upregulation of *PD-L1* through the induction of a transcription factor, interferon regulatory factor-1 (30). The present study identified CMTM6 and CMTM4 as a novel regulator of PD-L1 in dogs, although the detailed role of CMTM6 and CMTM4 in PD-L1 regulation remains unclear. The co-localization, molecular binding, and lysosome-mediated degradation of PD-L1 should be examined in canine cells to confirm the post-translational regulation exerted by CMTM6 and CMTM4. Although many other molecules could be involved in PD-L1 protein expression, this study will be an important step to fully understand the regulation mechanisms of PD-L1 in canine cancers.

In human NSCLC, a positive correlation between CMTM6 and PD-L1 expression was observed via immunohistochemistry. Moreover, the authors noticed that no samples without CMTM6 expression expressed PD-L1 (31). In this study, although both PD-L1 and CMTM6 expression was identified in the same cancer samples, their correlation remains obscure due to the limited sample size with no cancers lacking CMTM6 expression. Additional studies including a large number of samples, in addition to other cancer types, are necessary to clarify the importance of CMTM6 in the tumor microenvironment of canine cancers.

A recent study involving RNA-seq data sets revealed that *CMTM6* expression is correlated with poor prognosis in human gliomas, possibly due to the inhibition of T-lymphocyte-mediated antitumor immunity (32). In a mouse model, CMTM6-depleted tumor cells created by the introduction of short hairpin RNA tended to grow slower, resulting in the extended survival of tumor-bearing mice (15). These studies suggest that CMTM6 is a potential therapeutic target that might provide alternative options to PD-1/PD-L1 blocking antibodies. However, further exploration is necessary to identify CMTM6 inhibitors or a gene therapy targeting CMTM6, and their efficacies should be further investigated in preclinical models before clinical application. In this regard, given that CMTM6 function is conserved in dogs, dog patients could serve as a better animal model than mice for evaluating anti-CMTM6 therapy because naturally occurring cancers in dogs share similarities with human cancers in many aspects including their biological behaviors, responses to conventional treatment, genetic heterogeneities, and immune responses (33).

Taken together, our data provide the first evidence of CMTM6 and CMTM4 expression in canine cancers and their possible involvement in PD-L1 regulation. Further studies, both fundamental and in relation to clinical observations, are warranted to clarify the functions of CMTM6 and CMTM4 in dogs and to establish a better immunotherapy that targets the PD-1/PD-L1 axis through modulating CMTM6 and CMTM4 expression.

## Data Availability Statement

The raw data supporting the conclusions of this article will be made available by the authors, without undue reservation, to any qualified researcher.

## Ethics Statement

The animal study was reviewed and approved by #15-0149.

## Author Contributions

SK, SM, and KO designed and supervised the project. HT, NM, EM, YI, and AK performed the experiments. HT, SK, NM, EM, YI, AK, and TO analyzed the data. HT, SK, and NM prepared the manuscript. All authors reviewed and approved the manuscript.

## Conflict of Interest

The authors declare that the research was conducted in the absence of any commercial or financial relationships that could be construed as a potential conflict of interest.
