# Supplementary material for: Expression Analysis of Canine CMTM6 and CMTM4 as Potential Regulators of the PD-L1 Protein in Canine Cancers
Source: Front Vet Sci. 2020 Jun 11;7:330. doi: 10.3389/fvets.2020.00330 (PMC7300202; doi:10.3389/fvets.2020.00330)
Supplement: Supplementary file 1 [file Data_Sheet_1.pdf]

Supplemental Material

Materials and Methods

Flow cytometry

Binding specificity of the anti-PD-L1 mAb were assessed by flow cytometry. To this end, we used a canine PD-L1 (cPD-L1)-enhanced green fluorescent protein (EGFP) fusion protein-expressing Chinese hamster ovary (CHO) DG44 cell line. In order to establish a cell line stably expressing cPD-L1, the expression vector for the cPD-L1-EGFP fusion protein (pEGFP-N2-cPD-L1) (12) was introduced into CHO DG44 cells (Thermo Fisher Scientific, Waltham, MA, USA) using Lipofectamin LTX Reagent (Thermo Fisher Scientific) according to manufacturer's instructions. Stably expressing cells were selected in CD-DG44 medium (Thermo Fisher Scientific) supplemented with GlutaMAX supplement (20 mL/L, Thermo Fisher Scientific), 10% Pluronic F-68 (18 mL/L, Thermo Fisher Scientific), and 800 µg/mL of G418 (Enzo Life Sciences, Farmingdale, NY, USA). A cell line with high PD-L1 expression was isolated by using limiting dilution method. Next, the cPD-L1-EGFP-expressing CHO DG44 cells were incubated with 10 µg/mL of 6C11-3A11 at room temperature for 30 minutes. Cells were washed twice and incubated with allophycocyanin-labeled anti-rat immunoglobulin secondary antibody (SouthernBiotech, Birmingham, AL, USA) at room temperature for 30 minutes. Cell fluorescence was analyzed using a FACS verse flow cytometer (BD Biosciences). CHO DG44 cells transfected with empty pEGFP-N2 vector (Clontech, Palo Alto, CA, USA) were used as a negative control. Rat IgG2a (BD Biosciences) were used as isotype-matched control antibody. Phosphate-buffered saline (PBS) containing 10% normal goat serum (Sigma-Aldrich, St. Louis, MO, USA) was used as wash and dilution buffers. Singlet cells with high EGFP expression were gated and analyzed for antibody binding.

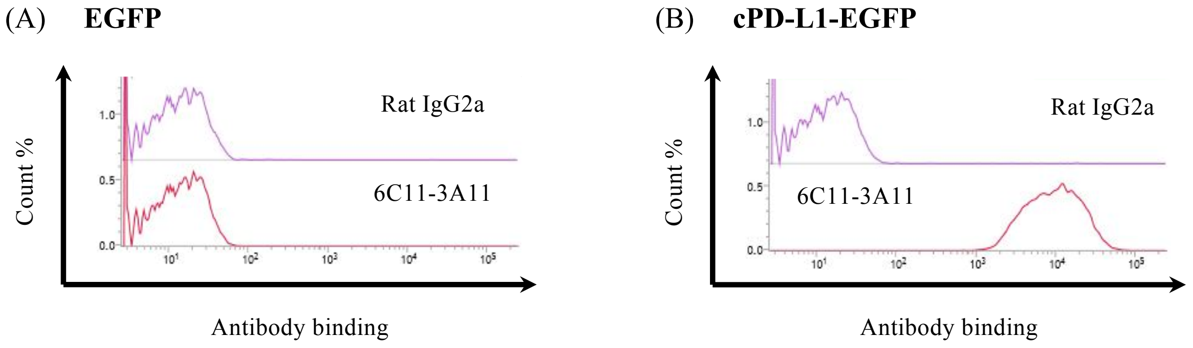

Figure S1. Binding specificity of anti-PD-L1 mAb 6C11-3A11 as assessed by using flow cytometry. Bindings of 6C11-3A11 or isotype-matched control antibody (rat IgG_{2a}) to EGFP-expressing (A) and cPD-L1-EGFP–expressing (B) CHO DG44 cells are shown.
